# Nanomembranes-Affiliated Water Remediation: Chronology, Properties, Classification, Challenges and Future Prospects

**DOI:** 10.3390/membranes13080713

**Published:** 2023-08-01

**Authors:** Divya Bajpai Tripathy, Anjali Gupta

**Affiliations:** Division of Chemistry, School of Basic Sciences, Galgotias University, Greater Noida 201312, India; anjali21in@gmail.com

**Keywords:** nanomembranes, nanofiltration, water remediation, nano-processing, ultrafiltration

## Abstract

Water contamination has become a global crisis, affecting millions of people worldwide and causing diseases and illnesses, including cholera, typhoid, and hepatitis A. Conventional water remediation methods have several challenges, including their inability to remove emerging contaminants and their high cost and environmental impact. Nanomembranes offer a promising solution to these challenges. Nanomembranes are thin, selectively permeable membranes that can remove contaminants from water based on size, charge, and other properties. They offer several advantages over conventional methods, including their ability to remove evolving pollutants, low functioning price, and reduced ecological influence. However, there are numerous limitations linked with the applications of nanomembranes in water remediation, including fouling and scaling, cost-effectiveness, and potential environmental impact. Researchers are working to reduce the cost of nanomembranes through the development of more cost-effective manufacturing methods and the use of alternative materials such as graphene. Additionally, there are concerns about the release of nanomaterials into the environment during the manufacturing and disposal of the membranes, and further research is needed to understand their potential impact. Despite these challenges, nanomembranes offer a promising solution for the global water crisis and could have a significant impact on public health and the environment. The current article delivers an overview on the exploitation of various engineered nanoscale substances, encompassing the carbonaceous nanomaterials, metallic, metal oxide and metal–organic frameworks, polymeric nano-adsorbents and nanomembranes, for water remediation. The article emphasizes the mechanisms involved in adsorption and nanomembrane filtration. Additionally, the authors aim to deliver an all-inclusive review on the chronology, technical execution, challenges, restrictions, reusability, and future prospects of these nanomaterials.

## 1. Introduction

Water is a precious resource essential for life, and access to clean water is a basic human right. Unfortunately, water contamination has become a global crisis, affecting the health and well-being of millions of people worldwide. Polluted water can cause a range of diseases and illnesses, including cholera, typhoid, hepatitis A, and dysentery, among others. The World Health Organization (WHO) estimates that contaminated water and poor sanitation are responsible for the deaths of approximately 3.4 million people annually, mostly children under the age of five. Conventional methods of water remediation, such as chemical treatment, flocculation, sedimentation, filtration and disinfection that generally involve chlorination, ozonation, and ultraviolet radiation have been exploited for many years to remove contaminants from water. However, these methods have several challenges, including limitations in removing specific contaminants, high cost, and environmental impact [1,2].

One significant challenge with conventional water remediation methods is their inability to remove emerging contaminants, such as pharmaceuticals, pesticides, and endocrine disruptors, which can have adverse health effects on humans and wildlife. These contaminants are often present in low concentrations, making their removal difficult with conventional methods. As a result, they may persist in water and accumulate over time, posing long-term risks to public health and the environment. Another challenge with conventional methods is their high cost. Chemical treatment methods, such as coagulation and flocculation, require large amounts of chemicals, energy, and equipment, which can be expensive and resource-intensive. Similarly, sedimentation and filtration methods require high maintenance and operational costs, which can be prohibitive in many settings. Conventional water remediation methods can have adverse environmental impacts. Chemical treatment methods, for instance, can produce toxic by-products, which can harm aquatic ecosystems and wildlife. Sedimentation and filtration methods can also generate large amounts of waste, which can be difficult to manage and dispose of safely [3].

Nanomembranes offer a promising solution to the challenges associated with conventional water remediation methods. Nanomembranes are thin, selectively permeable membranes, typically less than 100 nanometers in thickness, that can remove contaminants from water based on size, charge, and other properties. Nanomembranes offer several advantages over conventional methods, including their ability to eradicate emerging contaminants, their low operational costs, and their reduced environmental impact. Nanomembranes can remove incipient contaminants, such as pharmaceuticals and pesticides, with high efficiency. The small pore size of nanomembranes allows them to filter out even the smallest particles, including viruses and bacteria. This makes them highly effective in removing emerging contaminants, which are often present in low concentrations and difficult to remove with conventional methods.

Nanomembranes also have low operational costs. Unlike chemical treatment methods, which require large amounts of chemicals, energy, and equipment, nanomembranes require minimal energy and equipment. They also have low maintenance costs, making them an attractive option for water remediation in resource-limited settings. Nanomembranes have a reduced environmental impact compared to conventional methods. They produce less waste and require fewer chemicals, reducing their impact on aquatic ecosystems and wildlife. They also have a smaller carbon footprint, as they require less energy to operate [4].

In spite of having countless latent advantages, the field of nanomembranes still has several challenges that need to be addressed. There are also several challenges associated with the use of nanomembranes in water remediation [5]. 

One of the main challenges is fouling and scaling, which can occur when contaminants accumulate on the surface of the membrane, reducing its efficiency over time. The fouling of membranes takes place due to the presence of suspended solids (generally >0.01 microns), whereas scaling occurs because of the dissolved solids especially salts, when exceeded from their solubility. Fouling and scaling can be caused by several factors, including the quality of the feed water, the membrane material, and the operating conditions. Addressing fouling and scaling is critical for maintaining the long-term performance of nanomembranes. Fouling occurs when contaminants accumulate on the surface of the membrane, reducing its efficiency over time. Scaling occurs when mineral deposits accumulate on the surface of the membrane, leading to reduced water flux and enhanced pressure drop. Fouling and scaling can be caused by several factors, including the quality of the feed water, the membrane material, and the operating conditions. Fouling and scaling can be mitigated through several strategies, including cleaning and maintenance of the membrane, optimization of operating conditions, and the use of antifouling coatings. However, these strategies can be costly and time-consuming, and their effectiveness may depend on the specific application and membrane material [6,7,8,9,10]. 

Another challenge associated with the nanomembranes is cost-effectiveness. While nanomembranes have low operational costs, they can be expensive to produce, making them inaccessible in many settings. The cost of nanomembranes is mainly due to the high cost of raw materials, such as polymers and ceramics, and the complex manufacturing process required to produce membranes with nanoscale features [11]. 

Scientists and researchers are making continuous efforts to reduce the cost of nanomembranes through the development of more cost-effective manufacturing methods and the use of alternative materials. Exploration of the use of graphene and other 2D materials as alternative membrane materials is one of the recent examples, which may offer advantages in terms of cost, scalability, and performance [12].

An additional factor with nanomembranes is their potential impact on the environment. While nanomembranes have a reduced environmental impact compared to conventional methods, there are concerns about the release of nanomaterials into the environment during the manufacturing and disposal of the membranes. Nanomaterials may have unknown effects on aquatic ecosystems and wildlife, and there is a need for further research to understand their potential impact [13].

There are various challenges associated with the scale-up and implementation of nanomembranes in real-world applications. While nanomembranes have shown promise in laboratory settings, their performance and durability in real-world conditions are not well understood. Additionally, there may be regulatory and policy barriers that need to be addressed before nanomembranes can be widely adopted.

However, while nanomembranes offer several advantages over conventional methods in water remediation, there are also several challenges that need to be addressed. These challenges include fouling and scaling, cost-effectiveness, environmental impact, and implementation in real-world applications. Addressing these challenges will be critical for the successful implementation of nanomembranes in water remediation and for achieving the goal of providing access to clean water for all. 

This review article delivers an overview of the exploitation of various engineered nanoscale materials, including carbon-based nanomaterials, metallic nanomaterials, metal oxide-based nanomaterials and polymeric nano-adsorbents, MOFs (metal–organic frameworks) and nanomembranes for water remediation. The article also focuses on the mechanisms involved in adsorption and nanomembrane filtration. Additionally, the authors aim to provide a comprehensive review on the chronology, technical execution, challenges, restrictions, reusability and future prospects of these nanomembranes.

## 2. Chronology of Nanomembranes

The study of nanomembranes can be traced back to the effort of Langmuir and Blodgett, who created monolayers of omniphilic structures on the surface of water, which were then relocated onto the solid surfaces or grids [14,15]. In spite of noteworthy study, Langmuir–Blodgett (LB) membranes never became mainstream commercial applications, which was possibly due to the challenging synthetic procedures process involving single-layer development on liquid surfaces and transfer to solids. During the late 1970s, Sagiv created alkylsilane monolayers on silicon surfaces, giving birth to the concept of “self-assembled monolayers” (SAMs)—an arrangement of molecules on solid surfaces. This was a significant breakthrough, as it allowed for the formation of molecular films through controlled means. Sagiv also delved into the electrical conductivity of these SAMs, which is now seen as one of the first experiments in molecular electronics [16,17].

The formation of bifunctional organic disulfide monolayers on gold surfaces was first reported by Nuzzo and Allara in the 1980s [18]. They observed that by immersing a gold surface in a disulfide solution, a tightly packed molecular monolayer could be formed spontaneously within a few hours under normal conditions. The process for creating self-assembled monolayers (SAMs) was detailed in their study. The mechanism of thiol SAM formation on gold has been extensively researched and described in various publications and texts, including its structure, dynamics, and kinetics. When a gold surface is immersed in a thiol solution, the S–H bond in the thiol dissociates, releasing hydrogen and forming covalent Au–S bonds. The molecular backbones are then ordered laterally through intermolecular interactions, leading to the creation of well-ordered monolayers. This process is well documented and has been the subject of numerous reviews and books.

SAMs offer several benefits over Langmuir–Blodgett (LB) films, including ease of preparation and the ability to directly coat solid surfaces with regimented SAMs without the need for transmission procedures. Whitesides and his colleagues acknowledged the easiness of SAM fabrication as a significant benefit and extensively explored their potential applications. One prominent example is soft lithography, a surface-patterning technique that uses various printing methods to deposit SAM patterns on surfaces. This approach bridged the gap between physical chemistry and nanolithography, providing a simple and reachable approach to construct nanostructures on a chemical laboratory bench [19,20,21,22,23,24,25,26,27,28,29,30,31,32,33,34,35,36,37].

The layer-by-layer (LbL) technique for fabricating nanomembranes was introduced by Decher in the 1990s. To create a precise polymeric film with a thickness range of 15 nm to several hundred nm, a process called layer-by-layer assembly is utilized. This technique involves immersing a surface that carries an electrical charge into oppositely charged polyelectrolyte solutions, which leads to the formation of a defined polymeric film. This is achieved by the repeated deposition of alternating layers of positively and negatively charged polyelectrolytes, resulting in a well-controlled and highly ordered film. LbL membranes have found use in various applications, including surface protection and drug delivery. Another approach to generate carbon nanosheets is through the use of amphiphilic monolayers on a water surface. This method involves compressing floating films to create various molecular arrangements, leading to the formation of nanosheets. Stable nanosheets can be created by connecting the molecules in the films using covalent bonds, organometallic linkers, or crosslinking with UV exposure. To customize the properties of the nanosheets, factors such as the molecules exploited, surface tension, surface area, and linker or radiation exposure circumstances can be varied. By combining the Langmuir and Blodgett’s recognized procedures with new methods of linking molecules at the liquid–gas interface, promising possibilities have emerged for integrating these nanosheets into devices [38,39]. 

Geim and Novoselov made a significant discovery in the 2000s by exploring graphene, which is a nanomembrane only one carbon atom thick (0.35 nm). This material exhibited exceptional mechanical, electronic, and optical properties and has become the new benchmark for two-dimensional systems. Researchers have intensively studied graphene with the aim of introducing that into innovative technologies, including electronics, sensing, and medicine. However, the homogeneous and chemically inert surface of graphene posed a challenge to its efficient functionalization with other functional groups or biomolecules, which hindered its fast market entry [40,41,42,43].

## 3. Nanomembrane Production Methods

**Figure 1:**
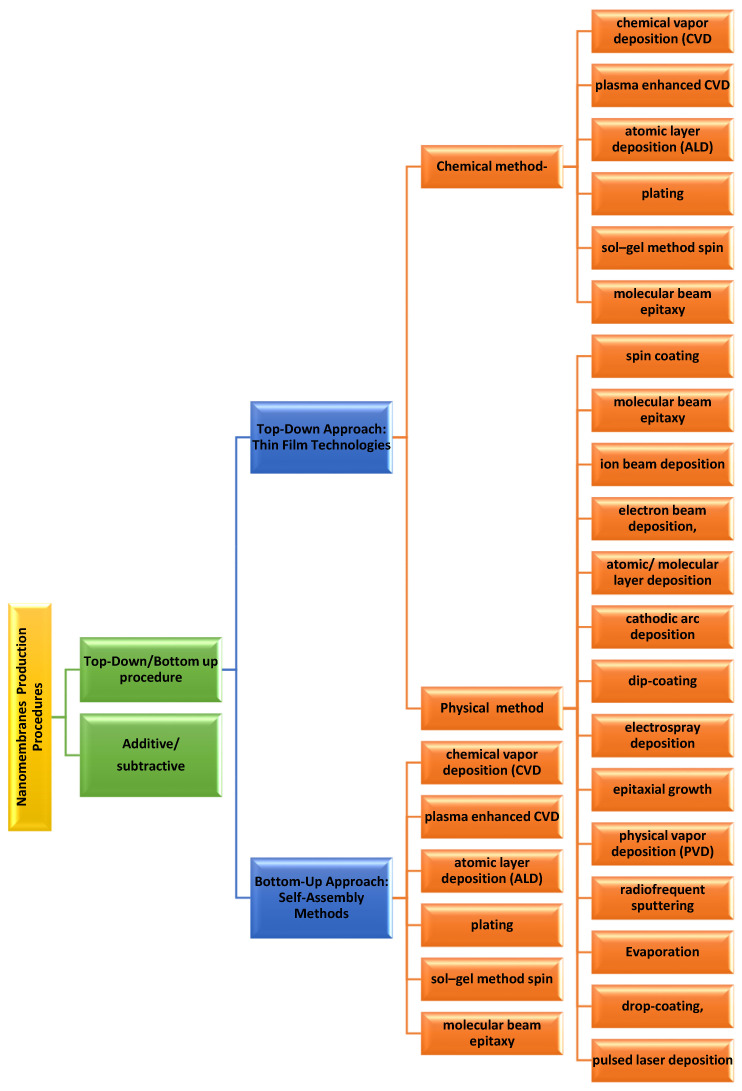
Classification of nanomembranes production process.

Nanomembrane production procedures encompass two distinct approaches. The first one is the additive/subtractive procedure of manufacturing the nanomembranes and another is the top–down/bottom–up tactic. The additive/subtractive fabrication is the procedure related with the membrane material. As the name indicates, in additive, new material has been incorporated to the membrane, whereas in subtractive, the material is detached from the membrane. The top–down/bottom–up approach explains in what way the addition and subtraction are achieved. Top–down or bottom–up approaches can be of either type, the additive or the subtractive. Details classification is provided in Figure 1 [44].

### 3.1. Top–Down Approach

Most of the ultrathin film deposition procedures involve the exploitation of microelectronics and microsystem (MEMS) technologies to produce biomimetic nanomembranes in addition to the combination with the sacrificial layer etching procedure. The top–down approaches are further divided into the physical and chemical types. Physical methods involve the deposition of nanomembranes in an optimized way that covers evaporation, radio-frequency sputtering, epitaxial growth, physical vapor deposition (PVD), spin coating, electrospray deposition, dip-coating, drop-coating, molecular beam epitaxy atomic layer deposition, ion beam deposition, electron beam deposition, cathodic arc deposition, molecular layer deposition, pulsed laser deposition, etc.

Chemical procedures encompass chemical vapor deposition, atomic layer deposition (ALD), plasma-enhanced CVD, plating, sol–gel method, molecular beam epitaxy, spin coating, etc. [45].

### 3.2. Bottom–Up Approach: The Following Are the Common Types of Bottom–Up Approaches [45]

#### 3.2.1. Self-Assembly Approach

Self-assembly can be understood as a natural phenomenon through which the structures organize themselves on their own into bigger units and the properties of larger units are regulated by the characteristics of smaller, as explained by the characteristics of their smallest component. 

#### 3.2.2. Langmuir–Blodget Method

This method exploits the self-assembly of surfactant molecules, which are specified to have a lipophilic tail and a lipophobic head moiety. Fatty acids, glycolipids and phospholipids are the common examples of this type. 

#### 3.2.3. Layer-by-Layer Self-Assembly

The layer-by-layer deposition adsorption procedure includes the deposition of alternate macromolecular layers with opposite charge over and over to provide 5 to 500 nm thick multilayers. 

#### 3.2.4. Block Copolymer Self-Assembly

This procedure involves the concurrent polymerization of two or more initial monomers to form a block copolymer with two or more dissimilar varieties of blocks, each consisting of a different homopolymer, and they are chemically dissimilar and immiscible. 

#### 3.2.5. Sol–Gel Process

In this deposition process, sol accumulates on a substrate and becomes steadily set into a gel with an incessant integrated solid network of nanoparticles and/or polymer in the liquid. 

#### 3.2.6. Dip-Coating

**Figure 2:**
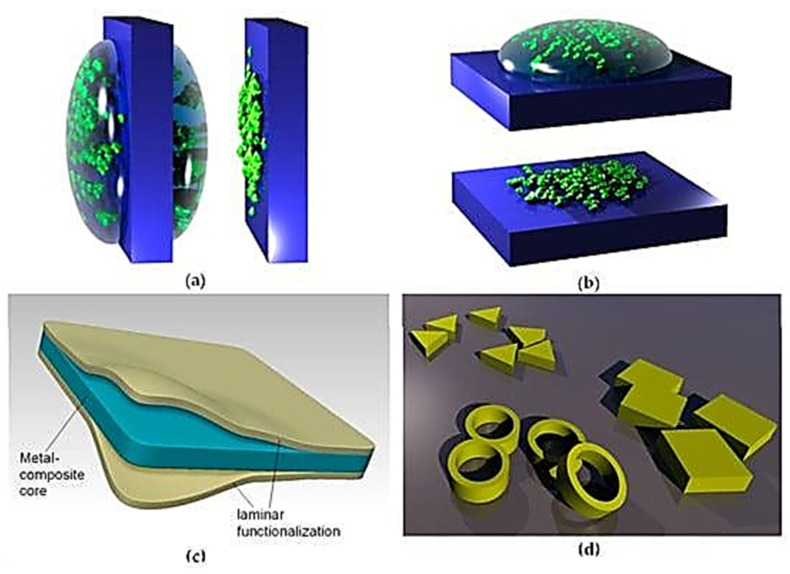
Nanomembrane production procedures: (**a**) Dip-coating; (**b**) drop-coating; (**c**) functionalization of synthetic nanomembrane by lamination; (**d**) nanotechnological zoo [Image reproduced with reference using open access work] [45].

In dip-coating, membranes form on an object after it is dipped in a solution containing the substance to be deposited. In an alternate way, nanoparticle suspension may also be exploited, as shown in Figure 2. 

#### 3.2.7. Drop-Coating

**Figure 3:**
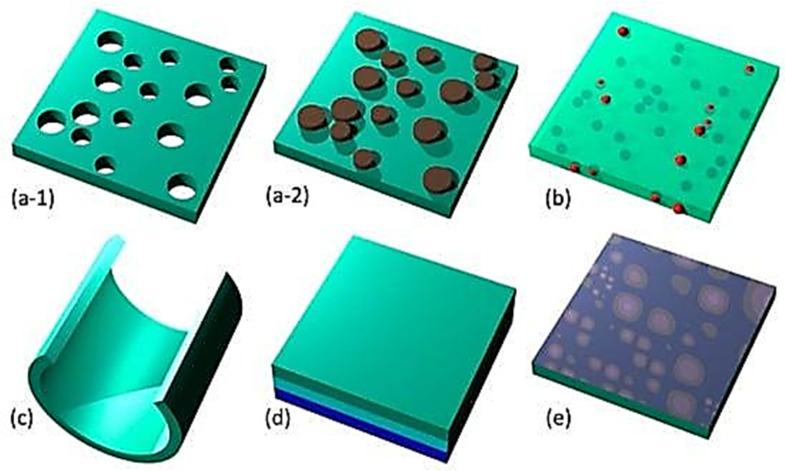
Nanomembrane functionalization. (**a**) Nanopatterning; (**b**) nanofillers; (**c**) rolled nanomembrane; (**d**) lamination; (**e**) surface activation [Image reprinted with reference using open access work] [45].

In drop-coating, droplets of the suspension or solution consisting of the substance to be placed are speckled on the object’s surface in precisely measured amounts. Figure 3 demonstrates the drop-coating procedure.

## 4. Types of Nanomembranes and Their Water Remediation Applications

**Figure 4:**
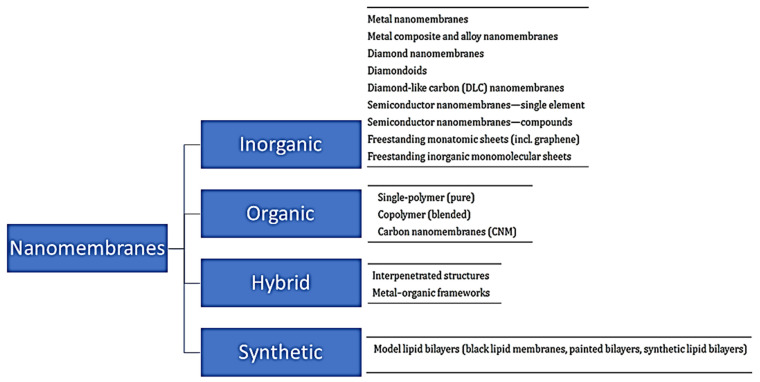
Broad classification of nanomembranes.

Nanomembranes are broadly classified into inorganic, organic and hybrid types and synthetic biomembrane types (Figure 4) based on the materials they are made from.

### 4.1. Organic Nanomembranes

Organic nanomembranes are made up entirely of one or more organic constituents that epitomize a significant group of existing self-supporting nanomembranes [45]. 

There is an enormous number of organic complexes and their blends that could potentially be exploited, although it is important to note that not all organic compounds are suitable for creating nanomembranes. Membranous structures cannot be made from certain organic compounds due to their gaseous or liquid state. Organic materials are composed of carbon compounds, except for pure carbon in either of its allotropic forms, as well as basic carbon compounds like carbides, oxides of carbon, carbonates, and cyanides. Many macromolecular/polymeric structures are well-suited for the production of freestanding nanomembranes, including polysaccharides, synthetic polymers, synthetic lipids, proteins, RNA, and DNA-based membranes [46,47,48,49,50,51].

Despite the potential advantages of macromolecular nanomembranes, these materials tend to exhibit a high level of sensitivity to temperature changes, and their beneficial characteristics are typically only maintained within a limited temperature range. They can also be attacked and dissolved by organic solvents and may be endangered by enhanced humidity. Furthermore, their mechanical properties tend to be impaired as membrane thickness reduces, and they typically have a low Young’s modulus. Most macromolecular nanomembranes start to slink and are enduringly plastically distorted underneath permanent pressure. Pyroxylin (nitrocellulose, collodion) was likely the first organic nanomembrane produced, which was created by Bechhold in 1907 [52]. Organic nanomembranes can be further classified into CNM, pure (single polymer) and blended (copolymer type). 

#### Carbon Nanomembranes 

The use of carbonaceous nanomaterials, encompassing graphene, carbon nanotubes (CNTs), carbon nanofibers (CNFs) and fullerenes, has gained widespread popularity in the field of environmental remediation. These nanomaterials possess distinct properties that make them ideal for numerous applications associated with air and water decontamination. Specifically, graphene and its oxide have been utilized as adsorbents for heavy metal ion removal. Furthermore, the occurrence of hydroxyl and carboxyl functional groups present on the graphene oxide surface enhances its sorption capacity. In summary, carbon-based nanomaterials have proved to be effective and versatile tools in the fight against environmental contamination [53,54].

**Figure 5:**
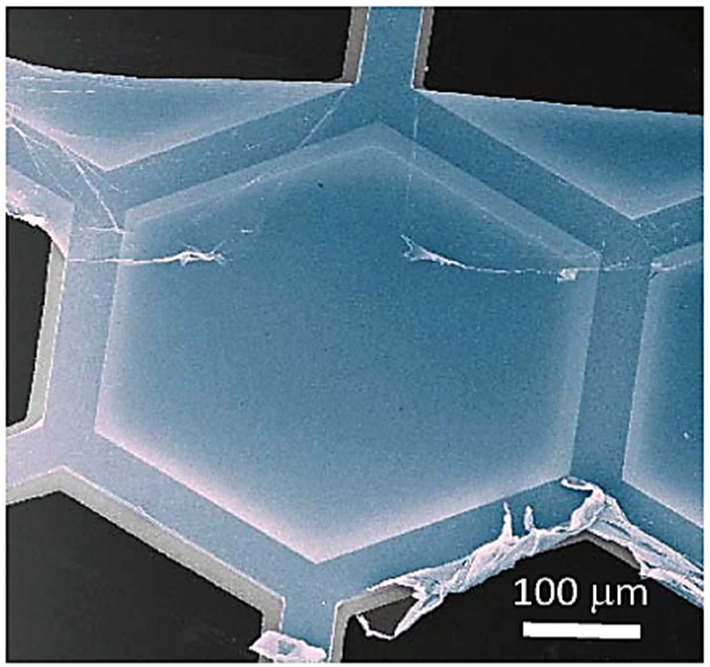
Image of CNM placed on hexagonal supporting group (reprint with copyright permission) [55].

Carbon nanomembranes (CNMs) are synthetic two-dimensional panes of carbon with unique physical and chemical characteristics that rely on their molecular composition, structure, and environment. With their molecular thinness, they act as “bulkless” interfaces that segregate various solid, liquid, or gaseous components while regulating the exchange of atoms and molecules among them. The thin and film-like nature of CNMs is evident in Figure 5, which depicts a He ion microscopy image of a CNM straddling a hexagonal gold mesh. To summarize, CNMs are ultrathin carbon-based sheets that can function as selective barriers for molecular exchange. The first CNMs were created around the time that graphene was discovered, but they did not receive as much attention initially. But as of now, it is very much clear that CNMs could offer a path to overcome some of the challenges associated with adapting graphene to various applications. CNMs have several advantages, including their thinness, chemical surface functionality, and ease of fabrication, which is similar to self-assembled monolayers (SAMs) and layer-by-layer (LbL) techniques. To create CNMs, molecules are arranged on a solid surface and crosslinked to produce a two-dimensional film. The resulting nanomembrane is then detached and released. The thickness, homogeneity, nanopore presence, and surface chemistry of the CNM depend on the original molecular monolayer. Due to their versatile fabrication and applicability, CNMs provide a robust foundation for applied research and new product development [55].

**Figure 6:**
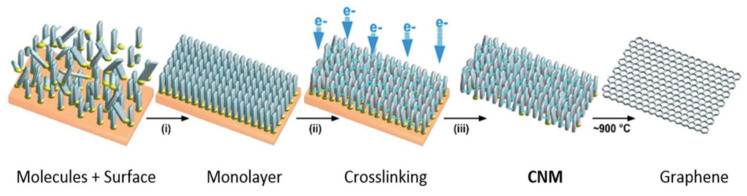
Fabrication route for CNMs and graphene: construction of self-assembled monolayers on a substrate (reprinted with copyright permission) [55].

Turchanin and Gölzhäuser (2016) [55] discussed various examples of the unique behavior of CNMs and discussed their potential applications in nanotechnology in their review, including filtration and sensorics. In this study, he claimed a more straightforward and universal approach to creating membranes that involved exposing a self-assembled monolayer on a solid surface to radiation, such as low-energy electrons or photons. This radiation exposure causes intramolecular bonds to break, resulting in the crosslinking of the rest of the molecular cores into a 2D framework. The result was the carbon nanomembrane (CNM) with a precise area, thickness, and surface functionality, as shown in Figure 6. This method of fabrication allowed for controlled functionalization by using self-assembled monolayers with specific functional moieties. A monomolecular SAM produces a CNM with even surface chemical groups, while a mixed molecular SAM generates a CNM with mixed surface characteristics. The mass density and defect density of the CNM are determined by the packing density and lateral order of the parenting SAM. The properties of CNMs were found to be highly diverse and claimed to be tailored to specific applications based on the environments through their manufacture, such as the molecular building blocks, SAM development, surface ordering, crosslinking, and release. These procedures can be modified to adjust the characteristics of the CNM. A CNM is essentially a molecular membrane without long-range order but with well-defined mechanical and electrical properties and surface functionalities. CNMs were found to be ideal for customization to meet specific application requirements. For instance, the exploitation of the molecular precursors with specific end groups leads to SAMs with even surface end groups, which, after crosslinking, resulted in a CNM with a specific surface functionality, which might not be the same as that of the SAM. In addition, amino-functionalized CNMs made up of nitro-functionalized aromatic SAMs have been exploited to attach different molecules such as polymers, dyes, and biomolecules to membrane surfaces, leading to a broader, fluorescent, or bio-functional CNMs.

Now, it can be concluded that carbon nanomaterials (CNMs) are versatile objects that can be easily fabricated and adapted to various environments. They possess exceptional durability against heat, electron irradiation, chemical substances, and pressure variations. By altering their precursors and treatment, CNMs can be made conductive or insulating. These materials are exploited in a broad range of applications, including microscopy support, pressure sensor diaphragms, gas and liquid filtration, protective coatings, and in conjunction with other 2D substances. CNMs possess such immense potential due to their 2D structure and effortless surface customization, making them ideal for crafting ultrathin membranes exploited in material separation and filtration. Conventional filtration membranes have a thickness of over 100 nm and operate through either a diffusion–solution process or via narrow pores. These membranes selectively separate a mixture of materials based on varying atomic or molecular species’ diffusion coefficients, resulting in the ideal infusion of specific species. This selectivity allows for the separation of gas or liquid mixtures into their individual constituents.

The diffusion process involved in materials separation through membranes can be slow, requiring high-pressure transformations among the two sides of the membrane to generate sufficient flux (φ). The formula for flux is given as φ = DAΔp/l, where D represents diffusivity, A represents the membrane area, Δp represents the pressure change between the two sides, and l represents the membrane thickness. Thin membranes need lower pressure differences compared to thicker ones to maintain the same level of flux. Two-dimensional membranes such as CNMs and graphene are considered the thinnest membranes and function like “sieves” in materials separation. The molecules can permeate through these membranes in a “ballistic” process, where they either translocate through a pore or bounce off the solid material between the pores. The unique potential of CNMs lies in their two-dimensional geometry and ease of surface modification. The primary application of CNMs is in the production of ultrathin membranes for materials isolation and filtration. To achieve this, the CNMs are altered to suit the specific separation or filtration requirements. Traditional membranes exploited for gas and liquid filtration are relatively thick (>100 nm) and require high-pressure differences to generate a sufficient flux. In contrast, thin CNMs require much lower pressure differences to maintain the same flux due to their “ballistic” permeation process. The emergence of mass-produced two-dimensional membranes may pave the way for CNMs to revolutionize the field of materials separation technology through disruptive innovation.

**Figure 7:**
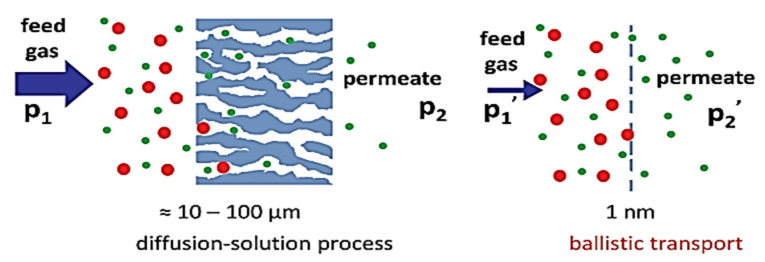
Conventional separation membrane and two-dimensional membrane (reprinted with copyright permission [55].

In their study, Ai and co-workers (2018) [50] investigated latent carbon nanomembranes (CNMs) for gas separation. They exploited a poly (dimethyl siloxane) (PDMS) composite membrane as a support to enhance the mechanical stability and reduce the roughness-induced strain. The scientists investigated the transportation of gases across PDMS membranes that were either left bare or covered with CNMs across a range of different gases. The results of this study revealed that while using a PDMS support with a single-layer CNM, the permeance values reduced to a range of 70% to 20% in comparison to PDMS without the CNM layer. In contrast, values between 4% and 2% are observed for three-layer CNMs, except for He and H_2_. The transport mechanisms for single-layer CNMs and multilayer CNMs are different. For single-layer CNMs, gas molecules flow through intrinsic pores or via direct transport through the 1 nm thick film, resulting in higher selectivity for H_2_/N_2_, He/N_2_, and CO_2_/N_2_. In contrast, it was suggested that the transport mechanism for CNM multilayers involves lateral diffusion between individual CNMs, resulting in higher permeation for He and H_2_ compared to larger gas molecules. They found that depositing a single layer of BPTCNM on PDMS enhanced gas selectivity for CO_2_/N_2_, indicating that the high selectivity was due to the great permeance of vertical channels for small molecules together with CO_2_. However, multilayers that had extra “tunneling” connecting the various layers exhibited reduced permeance and selectivity (see Figure 7). 

Pure (single polymer) and blended (copolymer type) nanomembrane:

Nanomembranes of this class consist entirely of organic materials and are typically made up of large macromolecules or polymers such as synthetic lipids, proteins, polysaccharides, RNA, DNA-based membranes, and synthetic polymers. These may be made up of a single polymer and known as pure (single polymer)-based nanomembranes and can be made up of two or more than two different polymer types and called blended (copolymer-type) nanomembranes. The creation of these nanomembranes involves using various types of polymers, including epoxy resins, polysulfone, polycarbonate, polyethersulfone, nylon, polyacrylate, polystyrene, cellulose, nitrocellulose, polyamide, polyimide, polypropylene, polydopamine, polyurethane, polyvinylchloride, poly (methyl methacrylate), polyester, poly(vinylidene fluoride), polytetrafluoroethylene (PTFE, Teflon), poly(lactic acid), polyacrylonitrile and polydimethylsiloxane (PDMS) [56].

Watanabe and Kunitake (2007) [57] successfully prepared a freestanding epoxy nanomembrane that was 20 nm thick and could be transferred intact onto a wire frame measuring 1 cm in diameter. Although the nanomembrane was too thin to be directly visible from a perpendicular position, light reflection was observed at an angle of 15. The high-quality epoxy nanomembranes did not show any visible defects or cracks. This study explored the feasibility of employing epoxy resins as a material for nanomembranes, which were subsequently transported onto AAO (anodized aluminum oxide) films for scanning electron microscopy (SEM) analysis. The thinnest freestanding nanomembranes demonstrated a consistent thickness of (23 ± 2) nm, as determined by averaging thickness values obtained from varied locations on the same sampling and dissimilar SEM specimens. These films exhibited flexibility on the uneven contour of the AAO support, which was only observed in films thinner than 40 nm. On the other hand, thicker films (80 and 200 nm) appeared stiff on the SEM support, but all membranes demonstrated flexibility on the macroscopic scale without any observable cracks or defects on the membrane surface. As such, the study concluded that the epoxy membranes were uniform and devoid of defects over their entire area. To evaluate whether the outstanding properties of epoxy resins remained consistent even at nanometer thicknesses, the mechanical strength of the thinnest membrane was examined through a bulging test. The tensile strength (r) and the ultimate elongation (e) were found to be 30 MPa and 0.2%, respectively. The value for r lies within the range of 1–100 MPa that has been established for conventional thick epoxy resins of various compositions. However, the eventual extension ratio was one order of magnitude smaller than the values for bulk epoxy resins. 

Polysulfone (PSU) is a popular membrane material due to its excellent mechanical, thermal, and chemical stability, making it suitable for manufacturing porous membranes for microfiltration (MF) to nanofiltration (NF). However, its low lipophobic properties make it susceptible to fouling during water purification. To overcome this issue, Ahmadipouya (2020) developed nanofiltration membranes by incorporating a metal–organic framework (MOF) into the polysulfone (PSf) matrix to remove organic dyes like methylene blue (MB), malachite green (MG), methyl red (MR), and methyl orange (MO) from water.

**Figure 8:**
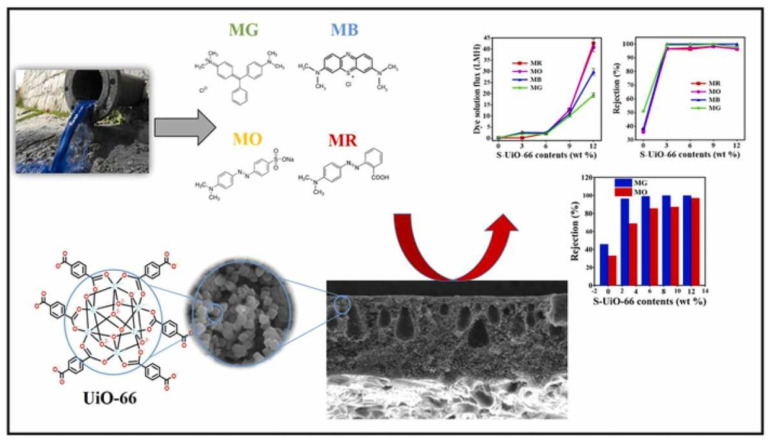
Nanofiltration membranes by the incorporation of UiO-66 metal-organic framework (reprinted with copyright permission) [58].

UiO-66 particles were exploited as lipophobic fillers to enhance the lipophobicity of the PSf membrane. These particles possess exceptional water stability, high thermal stability, chemical stability against organic solvents, and affinity toward organic dyes. The aperture size of UiO-66 MOF is about 6 Å, which is between the kinetic diameter of water molecules (≈2.6 Å) and most organic contaminants, making it a promising candidate for fabricating mixed matrix membranes (MMMs) for water purification. UiO-66 particles were synthesized using a solvothermal method and activated using two methods: Soxhlet extraction and centrifugation. The adsorption performance of UiO-66 MOF for the selected organic dyes was investigated, and the optimized UiO-66 MOF was incorporated into the PSf matrix to prepare the MMMs using the phase inversion technique (Figure 8). The prepared MMMs’ water remediation performance was evaluated using pure water flux and organic dye rejection, and their antifouling ability was assessed using Bovine Serum Albumin (BSA) solution as a sample. The results showed that the prepared MMMs exhibited improved lipophobicity, leading to reduced fouling and better permeation characteristics. Additionally, the MMMs’ organic dye rejection efficiency was significantly higher than that of the pure PSf membrane. The prepared MMMs also exhibited excellent antifouling characteristics when tested against BSA solution. A conclusion has been made in this study that incorporating UiO-66 particles into the PSf matrix to fabricate MMMs improved the lipophobicity, water remediation performance, and antifouling ability of the membranes. The study’s findings suggest that MMMs incorporating UiO-66 MOF could be a promising approach for developing high-performance membranes for water purification [58].

In another study by Abdelhamid and his co-workers [59], PSU has been investigated for ultrafiltration applications in the presence of clay nanoparticles, which enhanced the antifouling and flux recovery of the prepared membranes. Moreover, PSU has been modified with eugenol and zinc oxide to improve its ultrafiltration characteristics, antifouling, and antibacterial abilities. Quaternized graphene has been employed to reinforce PSU-based membranes for alkaline fuel cells. However, some polymer-based membranes are lipophilic, which makes them vulnerable to contamination by dyes and contaminants during wastewater remediation. The effectiveness and lifespan of membranes can be compromised by the obstruction of their pores, which can be caused by various factors including fouling. To address this issue, alternative materials are being explored to improve the lipophilicity and antifouling characteristics of membranes. Inorganic nanoparticles are a promising solution, with studies indicating that their incorporation into the membrane matrix during the phase inversion process can boost the membrane’s lipophobicity and antifouling characteristics. One such example is the use of titanium dioxide nanoparticles, which have been shown to enable the production of membranes with photocatalytic characteristics. 

Graphene is a two-dimensional material composed of a monolayer of atoms arranged in a honeycomb sp^2^ carbon lattice. Graphene boasts remarkable mechanical characteristics, chemical stability, and a significant surface area. Graphene-based membranes demonstrate characteristics akin to ceramic membranes and can be fashioned into films through graphene/graphene oxide fluid phase dispersions, mimicking polymers. Current research endeavors aim to elevate the transport characteristics of graphene-based membranes, particularly high permeability and selectivity for gases and liquids. Utilizing GO has shown to enhance forward osmosis and PSU anion exchange membranes’ performance. PSU nanofibrous membranes have also demonstrated additional antibacterial activity that make them more advantageous over others.

Graphene and/or GO can be functionalized to extend their efficiencies by grafting functional groups onto their surface. GO can attract functional groups and act as a potential adsorbent for removing heavy metal ions and organic contaminants. Fused heterocyclic compounds enriched with nitrogen, such as pyrano-pyrazoles, pyrazolo-pyridines, and pyrazolo-pyrido-pyrimidines, have shown expedient biological characteristics and can be exploited for functionalization. Some multicomponent reactions have been developed for synthesizing these heterocycles.

The performance of the filtration membranes is determined by their ability to reject salt or dye and allow water to pass through, posing a challenge to improve both characteristics without compromising the other. In this study, polymeric membranes based on PSU are prepared with the addition of f-GO. A heterocyclic compound loaded with nitrogen is attached to the surface of GO to functionalize it. The resulting membranes can be exploited for nanofiltration in water remediation applications and will be tested for their capacity to remove dyes with different surface charges, both anionic and cationic.

Amini and co-workers (2011) [60] investigated the efficacy of an ultraviolet radiation-treated acrylic grafted polysulfone nanomembrane for removing dyes from colored textile wastewater. In this study, the acrylic acid modification of the polysulfone ultrafiltration membrane was investigated, and the impact of various operating parameters on the modified membrane’s performance was evaluated. The outcomes of the study indicated that the membrane, which was subjected to photo grafting, demonstrated remarkable efficiency concerning both its flux and rejection capabilities. Specifically, the membrane exhibited a range of dye rejection rates between 86% and 99.9% and a hydraulic permeability of 7.6 L m^−2^ h^−1^ bar^−1^. However, the researchers observed that the rejection rates of dyes reduced as the salt concentration enhanced. This was attributed to a decline in the Donnan effect, which had a greater impact on low molecular weight and highly ionic dyes compared to other types of dyes. When 80 mM Na_2_SO_4_ was added to the dye solution, there was a reduction in dye rejection of over 15%. However, increasing the driving pressure from 1 to 4 bar did not significantly enhance rejection. The results of the study indicated that dyes possessing lower charges are more susceptible to operating pressure compared to those with higher charges. This suggests that the acrylic grafted nanomembrane could be a viable solution for removing dyes from colored textile effluent.

Numerous polymers, including polyethylene terephthalate (PET), polycarbonate (PC), cellulose nitrate, and allyl-diglycol-carbonate, have been thoroughly investigated for the preparation of ion-track membranes. These micro/nanofilters with nuclear tracks have found widespread application in diverse fields, such as microelectronics, biotechnology, fuel cells, air stream filtration, the pharmaceutical industry, biological cell separation, and wastewater recycling. By modifying the parameters of the irradiation process and chemical etching process, such as pore size, shape, and density, it is possible to create track-etched membranes with desired transport and retention characteristics. The crucial factors in pore creation are the etching temperature, pH, and time, which must be determined through experimentation. As ionizing particles traverse through polymers, they displace electrons and cause localized chemical alterations along their path. Permanent physical or chemical damage along the trajectory of a particle enables the chemically etched disrupted areas surrounding the particle path to erode faster than the undamaged material, resulting in visible tracks. This phenomenon is possible due to the higher track etching rate (Vt) in irradiated polymers compared to the bulk etching rate (Vb) [61,62,63,64,65].

Ziaie [66] disclosed the creation of nuclear track micro/nanofilters using polycarbonate (PC) films that are exposed to α particles from ^241^Am, which are trailed by chemical etching with an alkaline solution. This study also revealed that by increasing the etching time to a certain point, the pore diameters will also enhance, but for most cases, further increasing the etching time will cause the pore diameters to reduce. Eventually, at longer etching times, the pore diameters tend to become relatively constant. It has been observed that after 1 h of etching time at the same temperature, etching and annealing processes reach an equilibrium state. Therefore, to achieve the maximum pore diameter in the PC film, an etching solution with a normality of 4 N can be exploited for an etching time of 30 min. Increasing the temperature up to around 80 °C does not significantly affect the pore size. The standard deviation calculation of pore size measurement yielded an uncertainty of about 30%. It is possible to control the pore diameter by adjusting the etching parameters such as temperature, time and etchant solution concentration.

Wang and co-workers (2021) [67] discussed the utilization of the polystyrene nanomembrane photonic crystals for the detection of tetracycline through low triggered potential electrochemiluminescence and signal amplification. This application showcases the potential of utilizing photonic crystals in various fields such as medical diagnostics and environmental monitoring. This work claimed a novel electrochemiluminescence (ECL) strategy to detect the tetracycline antibiotic by exploiting gold-filled photonic crystals (GPCs) electrodes. The electrodes of GPCs are composed of photonic crystals formed by the self-assembly of polystyrene spheres and gold nanoparticles within the gaps of the crystals. These GPCs electrodes serve as a detection platform to bind antigens and label antibodies with Ru(bpy)_3_^2+^-COOH, which is a luminophore. The immobilized antigen on the surface of the photonic crystals is linked to Ru(bpy)_3_^2+^-COOH/Ab via immunoreaction to avoid direct contact with the gold nanoparticle surface. Electrochemiluminescence (ECL) emission is initiated by the electrochemical oxidation of tripropylamine (TPrA), since Ru(bpy)_3_^2+^-COOH cannot be directly oxidized on the electrode surface. TPrA^+^ cation and TPrA^·^ radicals, which are produced by TPrA oxidation, interact with Ru(bpy)_3_^2+^-COOH close to the electrode surface, leading to ECL emission. The electrodes exploited in GPCs consist of photonic crystals that form through the self-assembly of polystyrene spheres and gold nanoparticles in the gaps between the crystals. These electrodes are exploited as a detection platform to bind antigens and label antibodies with Ru(bpy)_3_^2+^-COOH, which is a type of luminophore. To prevent direct contact between the antigen and the gold nanoparticle surface, the immobilized antigen on the photonic crystal surface is linked to Ru(bpy)_3_^2+^-COOH/Ab through an immunoreaction. The electrochemical oxidation of tripropylamine (TPrA) initiates electrochemiluminescence (ECL) emission, since Ru(bpy)_3_^2+^-COOH cannot be directly oxidized on the electrode surface. When TPrA is oxidized, it produces TPrA+ cation and TPrA· radicals that interact with Ru(bpy)_3_^2+^-COOH near the electrode surface, resulting in ECL emission. The oxidation potential of TPrA (0.95 V vs. SCE) is lower than that of Ru(bpy)_3_^2+^-COOH (1.25 V vs. SCE), which results in a 300 mV lower ECL potential. By using nanomembranes made of photonic crystals, the electrochemiluminescence can be enhanced. To detect tetracycline antibiotic, a competitive immunoassay was conducted on GPCs electrodes using this technique, achieving a detection limit of 0.075 pg/mL (S/N = 3).

This demonstrates the potential for widespread application in the field of analysis and detection. The GPCs electrodes comprising photonic crystals, polystyrene spheres, and gold nanoparticles provide a sensitive detection platform that enables the detection of minute amounts of analytes.

In 2020, Nizam Uddin [68] conducted a study on sustainable freshwater harvesting from the atmosphere using nanocomposite fibers made of recycled polystyrene foams. The aim of this study was to develop cost-effective and efficient materials for water collection to address the water crisis in arid and semi-arid regions of the world. Plastic waste poses a growing environmental concern, and a sustainable solution to this issue is to recycle such waste into value-added materials. To this end, the current study employed the electrospinning technique to transform recycled expanded polystyrene (REPS) foam into super-lipophilic nanocomposite fibers by incorporating titanium dioxide (TiO_2_) nanoparticles and aluminum (Al) microparticles. These nanocomposite fibers demonstrated exceptional super-lipophilic characteristics, with a water contact angle of 152.03° and an effective fog-harvesting capacity of 561 mg/cm^2^/h. They have diverse industrial applications, such as water collection, filtration, tissue engineering, and composites. The recovered water can be utilized for drinking, agriculture, industrial and other purposes.

In a research work by Yang in 2011 [69], researchers utilized electrospun polystyrene nanomembranes to address contaminants such as methylene blue (10 mg/L), Cr^6+^ (5 mg/L), and Cu^2+^ (5 mg/L) found in simulated dyeing wastewater. The polystyrene liquor (8% (m/m) liquified in chloroform) was processed into a nanofibrous membrane with a diameter ranging from 250 nm to 15 µm, a detected pore size ranging from 3 nm to 0.5 µm, and a membrane thickness of 170 µm. A plate membrane system was utilized to examine the nanofiltration characteristics of the contaminants. The research demonstrated that the interception rates for contaminants were over 91% and the water flux ranged from 5.8 to 15.4 mL/(cm^2^h).

**Figure 9:**
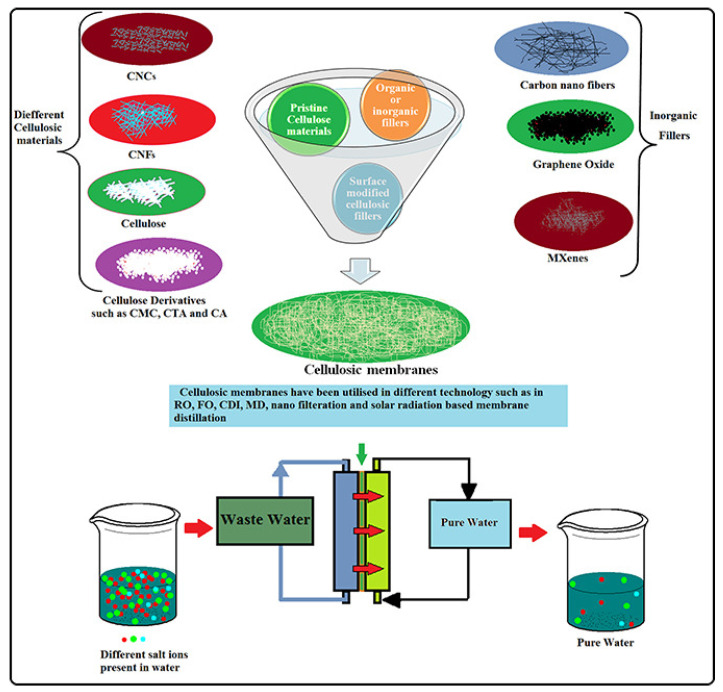
Water desalination using nanocelluloses/cellulose derivatives based membranes for sustainable future reprinted with copyright permission [70].

Highly efficient membranes for the desalination of seawater in various technologies, including FO, CDI, RO, MD, solar distillation, and electrodialysis have been successfully fabricated using nanocellulose and cellulose derivatives. Cellulose is a polymer that consists of multiple D-glucose moieties, and it is derived from renewable sources such as plants, wood, bacteria, and algae. This bio-polymer has been exploited in various forms, including macro and nano, and it can be treated with different chemicals to produce cellulose derivatives. These forms of cellulose have been utilized in the creation of water desalination membranes. Cellulose and their derivatives are a preferred material for membrane fabrication due to their attractive characteristics like biocompatibility, biodegradability, low cost, lipophobicity, and mechanical toughness (Figure 9). Cellulosic/cellulose-based membranes have been widely exploited as a sustainable solution for seawater desalination in the last decade. Meanwhile, commercial cellulosic membranes can achieve better permeability and a high level of salt or small molecule rejection with precise functionalities, porosity and pore size. Surface-tailored transport channels in cellulosic membranes have potential to provide opportunities for the recovery and separation of valuable products. Various types of cellulosic materials, including nanocelluloses (CNCs, CNFs, or BNC) and cellulose derivatives, have been utilized in the development of membranes for seawater desalination. These pristine or functionalized cellulosic membranes have been successfully applied in various membrane-based technologies, such as RO, FO, and MD, either as the membrane material itself or as a reinforcing agent to improve the performance of other membranes. While there are advantages to using certain materials, such as high biocompatibility and low toxicity, there are also drawbacks that must be considered. One of these limitations is their sensitivity to pH levels, which can impact their effectiveness. Additionally, these materials may exhibit reduced activity when subjected to higher temperatures, and they may not possess adequate thermal or mechanical strength. Furthermore, they may be prone to fouling and may not have sufficient resistance to chlorine [70].

**Figure 10:**
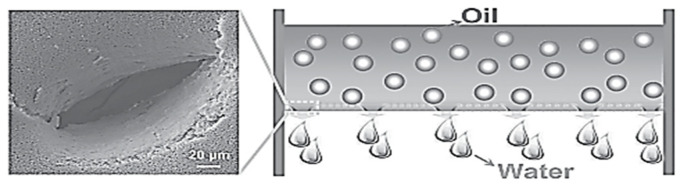
Programmed perforating process to fabricate dual-scaled porous NC membrane for oil/water separation Reprinted with copyright permission [71].

A dual-scaled porous nitrocellulose (NC) membrane with underwater superoleophobicity for highly efficient oil/water separation was fabricated by a facile perforating method (Figure 10).

The NC membrane is a commonly available material that has been functionalized and widely exploited in microfluidic technology, immunoassays, and biochemical analyses due to its excellent wetting characteristics and high protein-binding capability. In this study, researchers developed perforated nitrocellulose (p-NC) membranes with dual-scaled pores consisting of intrinsic nanopores and an array of perforated micropores. The p-NC membranes exhibited exceptional underwater superoleophobicity and high efficiency in separating oil and water. The micropores facilitated faster and easier water penetration through the membrane, with a water penetration time of only 8 min for 40 mL of water. In contrast, the NC membrane with only overlapped nanopores showed much slower water penetration, taking 103 min for 40 mL of water to pass through. The p-NC membranes were able to selectively and efficiently separate water from various oil/water mixtures, including gasoline, diesel, hexane, petroleum ether, and high-viscosity crude oil/water mixtures without the need for external power. The separation efficiency was greater than 99%. The separation time and intrusion pressure of the p-NC membrane for different oil/water mixtures could be easily adjusted by controlling the size of the perforated micropores. Additionally, the p-NC membranes demonstrated excellent underwater superoleophobicity in corrosive liquids, indicating excellent environmental stability and promising applications in practical oil spill cleanup and oily wastewater remediation. The p-NC membranes with dual-scaled pores and an array of perforated micropores have superior separation efficiency and stability, making them ideal for oil/water separation in various applications. The membrane’s perforated micropores can be tailored to achieve desired separation times and intrusion pressures, while the underwater superoleophobicity ensures high efficiency in separating oil and water [71,72].

Qi and co-workers conducted a recent study where they incorporated polydopamine (PDA) into ionic liquid-capped polyimide (IL-PI) membranes using an in situ growth method, resulting in a membrane with strong PDA adsorption characteristics. The IL-PI membranes were lipophobically modified with an IL containing lipophobic groups and PDA. The polymerization time was controlled to create a composite membrane that effectively separated oil–water emulsions. Scanning electron microscopy revealed an enhancement in PDA content in the composite membrane fibers and on the surface with longer PDA coating times. The PDA coating reduced the surface contact angle of the membrane from 72.87° to 12.06° and improved its wettability. The PDA-modified fibrous membranes exhibited an excellent separation of emulsified oil–water mixtures, achieving a maximum membrane flux of 280 L·m^−2·^h^−1^ and a separation efficiency of >99%. After ten repeated cycles, the separation efficiency remained >92%. This study presents a promising approach for designing future wastewater remediation solutions [73].

In one more research work, researchers investigated the water permeability of polyimide/GO thin film with a multilayer structure having an interlayer spacing of about 0.83 nm. The concentration of GO was between 0 and 0.02 wt. %, and the lipophobicity of the film enhanced with increasing GO concentration. The permeate water flux enhanced from 39.0 ± 1.6 to 59.4 ± 0.4 L/m^2^h under 300 psi with increasing GO concentration, while rejections of NaCl and Na_2_SO_4_ only slightly reduced from 95.7 ± 0.6% to 93.8 ± 0.6% and 98.1 ± 0.4% to 97.3 ± 0.3%, respectively. The interlayer spacing of GO nanosheets acted as a water channel and significantly impacted the water permeability [74].

A nanocomposite membrane of silver-doped fly ash/polyurethane (Ag-FA/PU) was successfully fabricated in a one-step electrospinning process, incorporating fly ash particles (FAPs). The process involved using a colloidal solution of polyurethane (PU) with FAPs and an Ag metal precursor, which was electrospun to create a spider-web-like nanocomposite membrane. The presence of N, N-dimethylformamide, a solvent of PU, reduced silver nitrate to Ag nanoparticles (NPs). The incorporation of Ag NPs and FAPs into the electrospun PU fibers was verified through electron microscopy and spectroscopic techniques. The addition of these NPs onto the PU nanofibers resulted in a spider-web-like nano-netting for NPs separation, enhanced absorption capacity for the removal of carcinogenic arsenic and toxic organic dyes, and antibacterial properties with reduced bio-fouling for membrane filter application. The Ag-FA/PU nanocomposite membrane exhibited promising potential for water remediation, demonstrating its cost-effectiveness and environmentally friendly nonwoven matrix for water purification. This approach offered a new opportunity for using one pollutant material to control other contaminants in a scalable and cost-efficient manner. Preliminary observations suggest that the Ag-FA/PU nanocomposite membrane is suitable for water remediation, making it a promising candidate for addressing water contamination issues [75].

In a study conducted by Asman [76], poly(vinyl pyrrolidone) (PVP) and dextran, which are water-soluble complex polymers, were utilized for the ultrafiltration (UF) of aqueous Fe^3+^ solutions utilizing poly(methyl methacrylate-co-methacrylic acid) (PMMA-co-MA) membranes. The study examined the impacts of polymer concentration and pH on the filtration of Fe^3+^ solutions as well as the volume collected and percentage retention (R%). The findings revealed that increasing polymer concentration resulted in reduced PMMA-co-MA membrane permeability, while pH enhanced Fe^3+^ solution retention. The retention rates for Fe^3+^ solutions with PVP and dextran were found to be 62% and 48%, respectively, at pH 3.0, for an 80 min filtration period, while the retention for Fe^3+^ solution without any complex-forming polymer was just 14%. The membranes were examined by AFM analysis and contact angle measurements [77].

Polytetrafluoroethylene (PTFE) is a highly desirable polymer for creating porous membranes to filter aggressive streams, even under severe temperature conditions, due to its exceptional chemical resistance and thermal stability. The lipophilic nature of PTFE membranes also suggests their potential application in a process called membrane distillation, which is increasingly being explored as an alternative and advantageous means of reverse osmosis treatment for concentrated and preferably warm solutions. Commercial PTFE porous membranes are typically produced using a complex process that involves mixing PTFE powder with a lubricant liquid. The resulting paste is then extruded in the form of a flat sheet or tube, which is then stretched and sintered to create a porous structure consisting of nodes and tiny interconnected fibrils. The pore size generally ranges from 0.1 to 2–3 μm, depending on the preparation conditions. However, flat sheet expanded membranes are usually thin and require bonding to a polyethylene or polypropylene support, such as a woven or nonwoven fabric, to improve handling and mechanical characteristics. Unfortunately, this also leads to the lower heat and chemical resistance of the final product. However, PTFE membranes are generally expensive due to the complex and time-consuming production process. Despite this, their unique properties make them a highly desirable material for various filtration applications, including aggressive streams and high-temperature environments. Researchers continue to explore new methods of producing PTFE membranes that offer improved properties and lower costs, with the aim of making them more accessible for use in a wider range of applications [78,79,80].

The concept of inducing roughness to achieve super-lipophilic surfaces through nanoparticle inclusion has been well established, but challenges with consistency and secondary contaminants need to be addressed. To potentially solve these issues, a super-lipophilic nanofibrous membrane was proposed by electrospinning a blended solution of polyacrylonitrile and lipophilic polydimethylsiloxane (PAN/H-PDMS) and undergoing a post-heat treatment process. The process of carbonization results in the creation of a hierarchically nano-rough surface on electrospun nanofibers due to the differential shrinkage between PAN and H-PDMS. This micro–nanoscale roughness significantly improves the super-lipophilicity of the material, with a water contact angle (WCA) of 163.48° and sliding angle (SA) of 4.2°. The resulting composite super-lipophilic nanofibrous membrane (CSN-M) exhibits excellent robustness against tape peel, abrasion, and bending cycles, maintaining a WCA higher than 158° and SA less than 6.5°. Additionally, the membrane displays a self-healing feature, which restores the WCA to 162.25° and reduces the SA to 5.0° after heat treatment at 60 °C. The CSN-M has a tensile modulus of 12.11 Mpa, a hydrostatic pressure of 39.18 cmH2O, and excellent breathability. It is highly permeable, durable, and strong, making it ideal for applications such as water/oil separation and self-cleaning [81].

### 4.2. Hybrid (Inorganic/Organic) Nanomembranes

**Figure 11:**
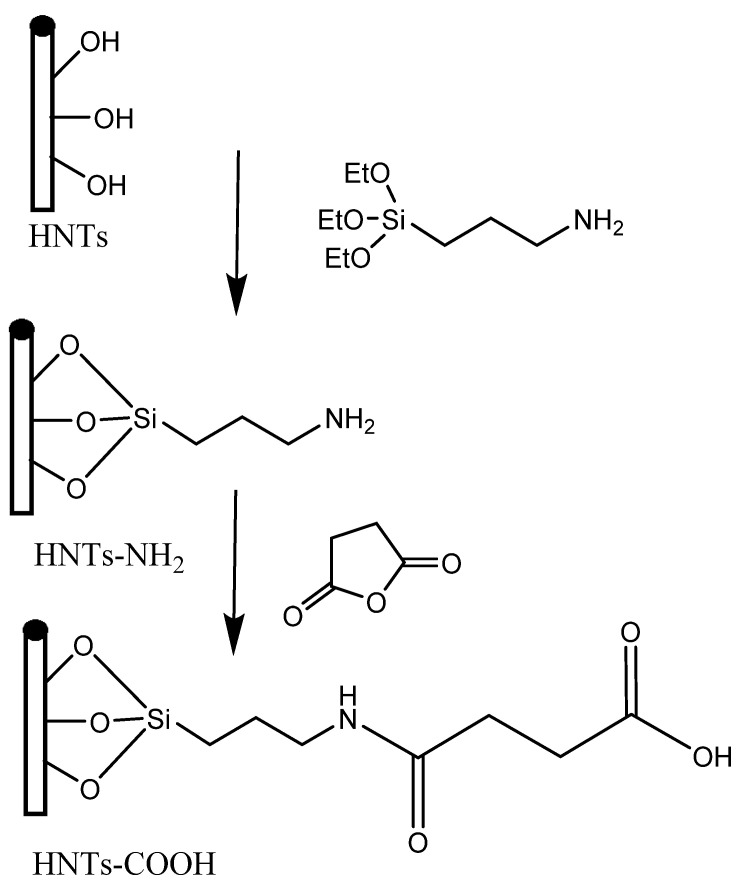
Synthesis of HNTs-COOH [82].

In addition to traditional inorganic carbon-based nanomaterials, transition metal-based nanomaterials and organic nanomaterials, there have been proposals to incorporate other nanomaterials for water remediation purposes. One such example is the use of magnetic halloysite nanotube (MHNT) composites that have been modified with molecularly imprinted polymers (MIPs) to selectively recognize and adsorb 2,4,6-trichlorophenol (TCP) for the remediation of wastewater. It has been suggested that this approach has potential for the development of commercially available products. In this work, the researchers developed a magnetic molecularly imprinted polymer (MMIP) for the selective recognition of 2,4,6-trichlorophenol (TCP) using magnetic halloysite nanotubes particles (MHNTs) as the base. Scientists have produced magnetic halloysite nanotubes (MHNTs) by attaching magnetic nanoparticles to carboxylic acid-functionalized halloysite nanotubes (HNTs−COOH) through a high-temperature reaction of ferric Tri acetylacetonate in 1-methyl-2-pyrrolidone (Figure 11). The researchers utilized the MHNTs to create molecularly imprinted polymers (MMIPs) and conducted several characterization techniques such as X-ray diffraction, Fourier transform infrared analysis, thermogravimetric analysis, vibrating sample magnetometer, transmission electron microscopy, elemental analysis, and Raman spectroscopy. The MMIPs had a 5.0−15.0 nm imprinted polymer film and demonstrated magnetic properties and thermal stability. Batch mode adsorption studies revealed the MMIPs’ specific adsorption equilibrium, kinetics, and selective recognition, with the Langmuir isotherm model fitting better than the Freundlich model. The MMIPs’ monolayer adsorption capacity was determined to be 246.73 mg g^−1^ at 298 K, and they exhibited high affinity and selectivity toward TCP over other phenolic compounds. Furthermore, the researchers found that the MMIPs were regenerable, with only a 11.0% loss in pure TCP solution and a 16.1% loss in coexisting phenolic compound solution after the fifth use. They also successfully used the MMIPs to remove TCP from environmental samples, highlighting the potential of MMIPs for the efficient removal of target contaminants from complex matrices. This study shows that the development of MHNTs and their utilization in MMIPs can lead to the effective removal of specific contaminants in complex samples [82,83].

**Figure 12:**
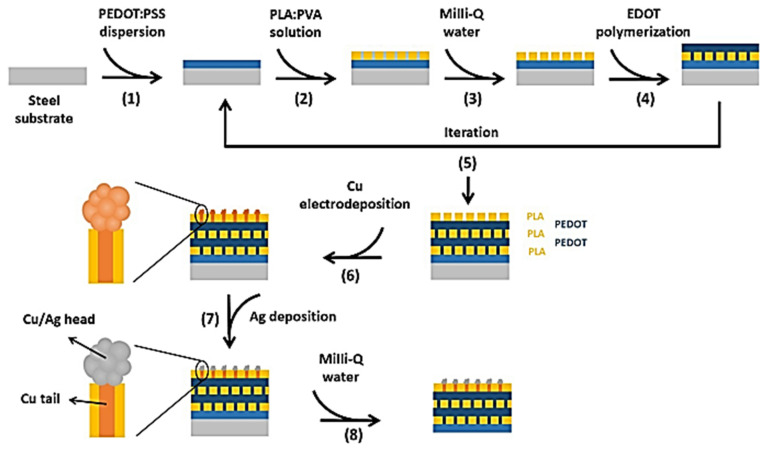
Fabrication procedure of the 5-PLA/PEDOT, 5-PLA/PEDOT-Cu and 5-PLA/PEDOT-Cu/Ag isolated nanomembranes. Image reprinted with reference using open access work [84] reproduced from open source under license http://creativecommons.org/licenses/by/4.0/ (accessed on 20 June 2023).

García-Torres et al., 2022 [84] developed a straightforward method for creating flexible electronic hybrid materials featuring nanostructured surfaces, using free-standing perforated two dimensional nanomembranes that host regimented 1D metal-based nanostructures. The fabrication process involves depositing alternating layers of perforated poly(lactic acid) (PLA) and poly(3,4-ethylenedioxythiophene), which are then incorporated with copper metallic nanowires (NWs) via electrodeposition. The top PLA layer with nanoperforations is then coated with silver through a transmetallation reaction (Figure 12). This approach combined the conformability and flexibility of the ultrathin, soft polymeric nanomembranes with the excellent electrical properties of metals, making it ideal for use in bio-integrated electronic devices. By tailoring the nanomembrane surface chemistry, this work demonstrated its sensing capabilities toward H_2_O_2_, with a good linear range (0.35–10 mM) of concentration, limit of detection (7 μm) and sensitivity (120 µA cm^−2^ mM^−1^). The hybrid nanomembranes produced were flexible and conformable, with selectivity toward H_2_O_2_, good stability and reproducibility, and such characteristics were confirmed by EDX, SEM, XPS, EIS, CV and contact angle analyses.

A simple fabrication procedure of epoxy resin and silica-based hybrid nanomembranes was also described by Watanabe 2009 [85]. In this study, the reaction of poly [(o-creyl glycidyl ether)-co-formaldehyde] (PCGF) and 3-aminopropyl triethoxysilane (APS) at room temperature was followed by spin-coating and baking at 120 °C to produce uniform nanomembranes with a thickness range of 20–50 nm. The epoxy and amine groups were homogeneously mixed through chemical linking, resulting in robust and defect-free nanomembranes. The nanomembrane had a lipophilic nature and persisted intact onto the water surface. It exhibited exceptional chemical steadiness without becoming swelled in many of the organic solvents, and its membrane morphology was preserved even after heating at 600 °C for 3 h, despite the complete eradication of the organic contaminant. The mechanical properties of the nanomembrane were not significantly altered from those of the epoxy-only nanomembrane described in an earlier study. The authors also discussed the significance of cross-linking density and hybridization in relation to the steadiness of various giant nanomembranes. This simple and straightforward method for fabricating hybrid nanomembranes can be applied to a wide range of precursor materials. This approach is expected to be highly effective for creating durable nanomembranes that are designed for specific applications. By carefully selecting the appropriate precursor materials and combining them in a hybridization process, a wide range of functional nanomembranes can be produced. The combination of macroscopic size and sub-100 nm thickness makes these nanomembranes suitable for use in highly competent material transport and extensive single-molecule governed devices for fundamental research. Additionally, these nanomembranes have potential practical applications in materials separation and specific ion transport. The formulated hybridization process elaborated in the article can help these nanomembranes perform well at higher temperatures [86].

### 4.3. Inorganic Nanomembranes

Inorganic nanomembranes have become gradually widespread recently for their latent use in water remediation applications. These membranes are typically composed of inorganic substances like metals, metal oxides and ceramics. One of the most promising inorganic nanomembranes for water remediation is graphene oxide. This material possesses excellent mechanical and thermal properties, as well as a high surface area, which allows for the efficient adsorption of contaminants. Other inorganic nanomaterials that have been investigated for water remediation include metal–organic frameworks (MOFs), zeolites, and mesoporous silica. MOFs, for example, are highly porous materials with tunable characteristics that can be exploited for the adsorption and degradation of organic contaminants. Inorganic nanomembranes have also been exploited to remove heavy metals from water. For example, iron oxide nanoparticles have been introduced to polymeric membranes to create nanocomposite membranes with improved performance in the removal of lead and cadmium from water. Inorganic nanomembranes show great promise in water remediation applications. With their unique characteristics and tunability, these materials have the potential to revolutionize the way we treat polluted water and confirm the availability of clean and harmless potable water for forthcoming generations.

Shayesteh and his co-workers (2016) [87] described the synthesis and performance evaluation of titania–gamma–alumina multilayer nanomembranes. The rejection ratio of a multilayered membrane in both acidic and alkaline solutions was investigated in a recent study. The support for the nanomembrane was prepared using the slip-casting method, which involved the use of alpha-alumina tubes. A gamma–alumina sub-layer and titania top layer were sequentially coated on the support, and the water flux and permeability of the nanomembrane were characterized. The study also examined the nanomembrane’s ability to reject microorganisms and several ions in a model wastewater at different pH levels. The support was evaluated for treating a model wastewater using a nanomembrane. The permeability of water through the nanomembrane was reduced when pressure in the range of 1–10 bar was applied. However, the permeability became almost constant at higher pressures and enhanced water flux. Rejection tests were conducted on a model wastewater containing ions, and the results indicated that the nanomembrane produced by the support fabricated using the slip-casting method could partially reject ions and successfully separate all microorganisms. Adjusting the pH was found to enhance ion rejection. The study exploited alpha–alumina tubes made through the slip-casting method as the support for the multilayered membrane, and the intermediate gamma–alumina sub-layer and titania top layer were sequentially coated on the support. The study also employed several characterization techniques to analyze the nanomembrane’s characteristics, including water flux, permeability, and ion rejection.

In a review, Cavallo and his co-authors [88] mentioned a method for creating inorganic nanomembranes (NMs): the two-step process involves depositing a thin active layer on a sacrificial layer, which is typically on a bulk substrate, followed by selective etching of the sacrificial layer to free the active membrane from the substrate. The functional layer can be deposited intentionally with strain or without, and its shape can be flat or conformed to different shapes. To create thin membranes, a multilayered structure is usually exploited, which involves a sacrificial layer between the functional membrane and the handle substrate. Group-IV NMs were observed to form thin sheets, rippled structures, and rolled-up structures through scanning electron micrographs. A trilayer configuration was exploited to balance strain in the growth direction and maintain the 2D geometry of the NM upon release. If anchor points remain, uniformly and compressively strained membranes relax by lateral expansion, resulting in periodic wrinkles. On the other hand, a high strain gradient in the growth direction causes the functional membrane to curl up and eventually form a tube [83].

Silicon-on-insulator (SOI) is a composite material that has gained widespread use in the semiconductor device manufacturing industry. It consists of a thin crystalline layer of Si, known as the template layer, that is separated from a bulk wafer by a SiO_2_ film. This technology was developed approximately 15 years ago, and it rapidly gained acceptance in the industry due to its ability to produce a thin Si template layer quickly and reliably. SOI has numerous advantages over bulk Si crystal, particularly in low-power circuit applications exploited in portable electronic devices. The use of a thin Si layer on top of an oxide has significantly improved semiconductor device performance. In addition to its applications in semiconductor device fabrication, SOI is the most commonly exploited platform for the development of micro- and/or nanoelectromechanical systems (MEMS and/or NEMS) and Si nanomembranes. In another case, the SiO_2_ layer that is buried acts as a layer that can be sacrificed, and it is removed through a selective etching process. Currently, commercially available SOI wafers have a top layer of Si that can be as thin as 20 nm. In the context of nanomembrane applications, these upper layers are often entirely detached and moved onto different host materials, although there are instances where contact points are retained. Hence, SOI has become an essential material in the semiconductor industry and has enabled significant advancements in low-power circuit applications and the development of micro- and/or nanoelectromechanical systems [89,90,91,92].

Yin and co-workers (2012) [93] proposed a method for enhancing the lipophobicity of thin film nanocomposite (TFN) membranes through integrating SiO_2_ nanoparticles (NPs) using an in situ interfacial polymerization procedure. They found that the improved solubilization and diffusion of water through the membrane contributed to the enhanced lipophobicity. A higher declining interfacial contact angle of liquid–vapor was suggested as the critical factor controlling the membrane surface lipophobicity, as previously shown in studies by Wang and co-workers (2011) [94]. Wang and colleagues suggested that depositing a polymer electrolyte membrane (PEM) on a rough substrate coated with sub-micrometer-scale silica spheres could result in a Wenzel state of membrane wetting, allowing high hysteresis contact angles of the liquid–vapor interface to be achieved. Sabir and co-workers (2016) [95] exploited the thermally induced phase inversion separation (TIPS) procedure to synthesize a polymer matrix SiO_2_ NP (PM-SNP)-conjugated membrane. Incorporating 0.4 wt% SiO_2_ NPs (PM-S4) into the PM membrane led to a marked improvement in salt rejection during reverse osmosis (RO) (flux of 2.39 L/m^2^h) compared to the unmodified membrane (flux of 2.1 L/m^2^h) due to the improved surface roughness conditions that facilitated water transport. Ahmad and colleagues (2015) [96] investigated the effect of SiO_2_ nanoparticles (NPs) at different weight percentages (1–5 wt%) on cellulose acetate and cellulose acetate/polyethylene glycol (CA/PEG) membranes. They observed that the inclusion of SiO_2_ NPs upgraded the thermal and mechanical steadiness of the CA/PEG membrane, resulting in an enhancement in flux from 0.35 to 2.46 L/m^2^h and an 11.41% enhancement in salt rejection. Among the tested membranes, CPS-5, containing 5 wt. % silica, was the most effective and resistant to fouling during RO. Pang and Zhang (2018) [97] developed a lipophilic fluorinated SiO_2_ NP-based thin-film nanocomposite (TFN) membrane for treating high salt content samples (2000 ppm). They observed an increase in desalination from 96% to 98.6% with a lowering down in flux from 0.99 to 0.93 m^3^/m^2^/day. Incorporating SiO_2_ nanoparticles into membranes can significantly enhance their performance in salt rejection and water transport. The improvements in thermal and mechanical stability, resistance to fouling, and salt rejection suggest that SiO_2_ NP-based membranes are a promising technology for various applications, including water remediation and desalination.

In one more study, researchers enhanced a glass fiber membrane by incorporating SiO2 nanoparticles and then subjected it to surface fluorination and polymer coating to develop an omniphobic membrane that can be exploited for the direct contact membrane distillation (DCMD) of a sodium lauryl sulfate (SLS) solution. The omniphobic membrane was pitted against a commercial polytetrafluoroethylene (PTFE) membrane, and comparisons were made based on contact angle and DCMD applicability. The results showed that the omniphobic membrane performed better when tested with various types of feed solutions like humic acid, kerosene oil, diiodomethane and detergent—for example, sodium lauryl benzene sulfonate—as compared to the PTFE membrane. Additionally, the omniphobic membrane exhibited anti-wetting characteristics toward water, ethanol, mineral oil and decane, while the PTFE membrane only displayed effectiveness when dealing with water [98].

In a study by Huang and co-workers, 2017 [99] a super-amphiphobic membrane was created for membrane distillation (MD) using electrospinning, calcination, and fluorination. The researchers found that this membrane showed better efficacy than a commercially available polyvinylidene fluoride (PVDF) membrane in treating concentrated feed solutions containing surfactants due to its super-amphiphobic feature. Efome and co-workers (2015) also prepared a SiO_2_-based anti-wetting super-amphiphobic membrane using a phase inversion immersion precipitation procedure to prepare PVDF/SiO_2_ flat sheet composite membranes for vacuum membrane distillation (VMD). The researchers studied the blending of super-lipophilic SiO_2_ nanoparticles with a PVDF-doped solution, and the modified membrane showed enhanced flux from 0.7 to 2.9 kg/m^2^h with a desalination rate of 99.98% in a VMD procedure. The addition of SiO_2_ nanoparticles and surface modification with fluorination and polymer coating showed promise in creating anti-wetting and super-amphiphobic membranes for DCMD and MD processes. These modified membranes displayed better performance than traditional commercial membranes in terms of contact angle and treatment efficiency against various feed solutions [100].

TiO_2_ is another coating material with favorable characteristics, such as nontoxicity, stability, low cost and photocatalytic characteristics, as noted by several authors [100]. Recently, CA/PEG membranes were modified with TiO_2_ in various loadings and exploited in RO and MD. Shafiq and co-workers (2018) [101] found that CA/PEG membranes with TiO_2_ loaded with 5, 10, 15, 20, and 25 wt% showed maximum desalination rates of 80, 90, 95.4, 85, and 80%, respectively, these results confirmed that the optimal loading was 15 wt% for maximum desalination. Membranes coated with TiO_2_ and exposed to UV radiation exhibited enhanced lipophobicity and self-cleaning characteristics. However, too much TiO_2_ blocked membrane pores and reduced membrane performance. Emami and co-workers (2018) and Stan and co-workers (2019) [102,103] observed that TiO_2_ NPs-coated membranes had exceptional self-cleaning characteristics under ultraviolet irradiation. Kwak and co-workers (2001) [104] conducted a study that revealed a TFC membrane, modified with TiO_2_ consisting of organic/inorganic hybrids, was less susceptible to fouling as compared to the pure PA membrane when exploited in RO. Safarpour and co-workers. (2015) established a TFN-RO film using interfacial polymerization and coating with reduced graphene oxide/TiO_2_. The altered membrane demonstrated improved lipophobicity and anti-fouling characteristics compared to the unmodified membrane [105]. Ren and co-workers (2017) created a TiO_2_-coated PVDF electrospun nanofiber membrane (ENM) that exhibited high flux (73.4 L/m2h) and salt rejection (99.99%) [106]. Various methods have been developed for producing super-lipophilic membranes, including nanomaterial-based membrane surface coating, dip-coating and post-modifications of virgin membranes [107].

Zinc oxide (ZnO) nanoparticles (NPs) have gained popularity as an additive due to their low price, high stability (physical, chemical, mechanical, and thermal), high surface area, surface functionalization, and remarkable antimicrobial and anti-corrosive characteristics. In membrane modification, ZnO has been shown to enhance the lipophobicity of blended membranes, which improves permeability and fouling resistance [103]. For example, ZnO NPs were incorporated into a cellulose acetate (CA) membrane via electrospinning to improve its antibacterial property for reverse osmosis (RO) [104]. Similarly, in order to reduce biofouling in membrane distillation (MD), ZnO was incorporated into cellulose acetate (CA), which proved to be an effective super-lipophilic/omniphobic membrane modification. Researchers prepared a composite membrane of polytetrafluoroethylene (PTFE)/poly(vinyl alcohol)/ZnO through electrospinning, which enhanced the contact area among the surface and microbes without agglomerating the nanoparticles. They also exploited PTFE /ZnO films for the self-cleaning of a fouled film during vacuum membrane distillation (VMD). The membrane demonstrated high chemical and thermal stability, with efficient salt rejection (99.9%) and eradication of dye (45%) as confirmed by photodegradation experiments. ZnO nanoparticles were also exploited to modify a glass fiber membrane via chemical bath deposition to produce an omniphobic film for DCMD (direct contact membrane distillation). This omniphobic film was exceedingly resilient to wetting through low-surface-tension liquids during DCMD and maintained a contact angle of 152.8° throughout the operation with a flux of 30 L/m^2^h and a salt rejection of 99.99% [108,109,110].

### 4.4. Synthetic Biological Nanomembranes

Model lipid bilayers are the examples of this class, the synthetic organic nanomembranes, which represent replicas of living nanomembranes [44]. The first model lipid bilayers were successfully synthesized in 1962 [111]. Initially known as “black lipid membranes” or “painted bilayers”, they were created as platforms for studying membrane processes in vitro, aiming to facilitate the analysis of transmembrane mechanisms and ion channel function. Among the early achievements in synthetic ion channels, tetra-substituted β-cyclodextrin was the first fully synthetically produced ion channel reported as early as 1982 [112]. This marked a significant advancement in the field, showcasing the potential to artificially create functional channels that mimic natural ion channels found in biological membranes. Subsequently, research in synthetic lipid bilayers and ion channels has continued to progress, leading to further insights into membrane biophysics and their applications in drug delivery, biosensing, and understanding cellular processes. The study of model lipid bilayers and synthetic ion channels remains a critical area of research in biophysics and nanotechnology.

## 5. Characteristics of Nanomembranes Attributed with Water Purification

Compared to larger-scale materials, nanoscale materials have a significantly larger surface area and demonstrate unique magnetic, optical, and electrical characteristics. When incorporated into membranes, they create structures with refined filtration mechanisms and diverse physical, chemical and biological characteristics. Some of the characteristics are as follows [113]:

### 5.1. Electrical Properties

**Figure 13:**
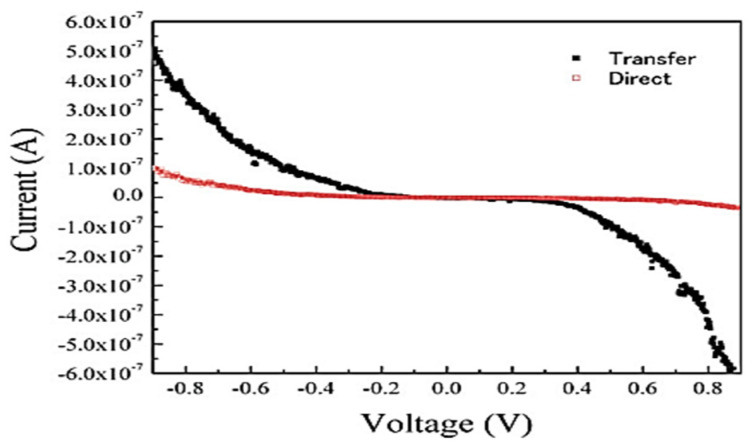
Output leakage curves of 30 nanometer thickness PCGF-PEI nanomembrane (reprinted with copyright permission) [57].

Electrical characteristics of nanomembranes were investigated using a potentiostat/galvanostat system, which measured the output leakage current of approximately 90 µA at 0.5 V for a 30 nm thick nanomembrane transferred onto a substrate. The electrical resistivity was calculated to be 0.5 × 10^11^ Ωcm (Figure 13). This value was found to be only seven times smaller than the value measured for a PCGF-PEI film directly fabricated onto a substrate (3.79 × 10^11^ Ωcm), indicating that the insulating behavior was not lost upon detachment from the substrate. The high insulating character of the nanomembrane suggested a defect-free behavior. The electrical resistivity value of the nanomembrane was essentially the same as that of a conventional bisphenol-A-type epoxy resin (10^10^–10^12^ Ωcm), which was claimed to be highly compatible with many chemical substances and had been exploited to develop superior functional and structural composites. The electrical properties of the nanomembranes were also found to be highly insulating, indicating a defect-free behavior. The resistivity value of the nanomembrane was comparable to that of a conventional bisphenol-A-type epoxy resin, suggesting that the resistivity value remained essentially the same when the material was prepared as a nanomembrane. These findings have important implications for the development of functional and structural composites using nanomembranes. Conclusions have been made that epoxy resins can be utilized as a material for nanomembranes. The resulting membranes were shown to be uniform, defect-free, and flexible, with a consistent thickness of (23 ± 2) nm. Moreover, the thinnest membrane exhibited a tensile strength that is comparable to conventional thick epoxy resins, while its ultimate elongation was substantially lower. These findings provide valuable insights for the development of new applications for epoxy resins in the field of nanotechnology.

### 5.2. Adsorption

Certain nanomaterials (NMs) grounded on nanostructured graphene, metal oxides, carbon nanotubes (CNT), zeolite, porous BN and electrospun nanofibers are capable of serving two functions, namely adsorption and the membrane filtration of heavy metal ions, phosphates and nitrates. These NMs possess active sites and high porosity, making them ideal for adsorbing contaminants. Electrospun nanofiber-based membranes that contain NMs exhibit intriguing characteristics for removing trace quantities of contaminants from water through filtration and adsorption, which is due to their porosity and large surface area. The adsorption of contaminants from an aqueous solution by these materials can occur through chemical binding, physical adsorption (caused by porosity, van der Waals attraction and the large surface area of NMs), or electrostatic attraction [114].

### 5.3. Photocatalysis

The photocatalytic characteristics of TiO_2_ NP-based NEMs are distinctive and include photodegradation and photoinduced super-lipophobicity. These characteristics provide the membrane surface with fouling resistant, antimicrobial and self-cleaning characteristics. The excitation of valence electrons of the photocatalyst occurs under UV light, causing their migration and resulting in the observed effects [115].

### 5.4. Antimicrobial Activity

The use of Silver NPs as antimicrobial mediators for NEM is widespread because of their highly effective biocidal characteristics. Silver intermingles with biochemical compounds, including cysteine, containing thiol groups (S-H) that contain phosphorus and sulfur. Through the formation of S-Ag or di-sulfide bonds, silver deteriorates the microbial proteins, denatures the DNA, and interjects the electron transport system, leading to its biocidal effect. In addition to silver, other nanoparticles such as Cu, CNT, and graphene have also been utilized for modifying commercial membranes to enhance their biocidal efficiency and application duration. These modified membranes deliver effective water disinfection while maintaining a high flux recovery ratio [116].

### 5.5. Chlorine Resistance

Functional nanomaterials such as zeolite and silica are being researched for use in NF and RO membranes due to their promising characteristics. Incorporating GO, SiO_2_, CNT and zeolite nanoparticles (NPs) into the barricade sheet of TFNCM has been shown to enhance the membranes’ resistance to chlorine. MWCNTs, in particular, act as a defensive coating for PA against free chlorine attack. Meanwhile, the improved chlorine resistance of GO-based membranes is principally attributed to the creation of hydrogen bonds between GO and PA that hinder the chlorine’s collaboration with active N-H bonds in PA. Zeolite-based membranes have also been explored for desalination as they are chemically robust and favorable. Zhu et al. [80] conducted a study on the steadiness of MFY-type zeolite films against chlorine cleaning and found that they maintained high chlorine stability. The zeolite membranes retained their high rejection of ions such as Mg^2+^ (90%) and Ca^2+^ (82%), and they also showed good rejections for Na^+^ (70%) and K^+^ (78%) after exposure to a hypochlorite cleaning solution (1000 ppm) for 7 days at an applied pressure of 3 MPa, with no noteworthy alteration in water flux or salt rejection [117].

## 6. Challenges with Nanomembrane-Enhanced Water Remediation

Nanomembranes have emerged as a potential solution for wastewater remediation due to their unique characteristics, but there are several challenges that need to be addressed. These challenges include the lack of information about the nanomaterials, their potential adverse effects on human health and the environment, and the need for effective and sustainable wastewater remediation methods. The rapid commercialization of nanomembranes has led to an enhancement in their production globally, but they also face various challenges that must be addressed to realize their full potential. This section outlines some of the key challenges in the application of nanomembranes for water treatment. Nanomembranes are susceptible to fouling, where contaminants and particles accumulate on the membrane surface or within its pores over time. Fouling can reduce filtration efficiency and increase operating costs, necessitating frequent cleaning or replacement. Scaling up nanomembrane production to meet large-scale water treatment demands can be challenging and costly. Developing cost-effective manufacturing methods without compromising performance remains a significant obstacle. They are vulnerable to mechanical and chemical degradation, impacting their stability and lifespan. Ensuring durability and longevity under varying water conditions is crucial for practical applications. Achieving high selectivity for target pollutants while avoiding interference from other compounds can be challenging. Fine-tuning membrane properties to selectively capture specific contaminants requires careful material design and engineering. The use of nanomaterials in water treatment raises concerns about potential environmental and health risks. Meeting stringent regulatory requirements and demonstrating the safety of nanomembrane technology is essential for widespread adoption.

Moreover, the performance of nanomembranes can be influenced by the complex and diverse composition of water sources. Variations in water chemistry may impact membrane stability, fouling rates, and contaminant removal efficiency. Some nanomembrane processes may require significant energy inputs for effective water remediation. Developing energy-efficient systems to minimize operational costs and reduce the environmental footprint is a critical challenge. Developing scalable and reproducible manufacturing techniques for nanomembranes is essential for cost-effective production and large-scale implementation. Integrating nanomembranes into existing water treatment systems and infrastructure can present compatibility challenges and require adaptations to ensure seamless operation.

Despite these challenges, ongoing research and technological advancements hold the promise of overcoming these obstacles. Collaborative efforts between researchers, industries, and regulatory bodies are essential to address these challenges and unlock the full potential of nanomembranes in providing sustainable and efficient solutions for water remediation. By addressing these concerns, nanomembranes can contribute significantly to mitigating water scarcity and ensuring access to clean water resources for a sustainable future [118,119,120,121].

## 7. Conclusions

Nanomembranes have emerged as a highly promising technology in the field of water remediation, offering innovative solutions to tackle the growing global water crisis. These ultrathin synthetic structures have demonstrated remarkable potential in various water treatment applications, including filtration, desalination, and contaminant removal. The development of nanomembranes has provided a breakthrough in water purification processes. Their nanoscale porosity allows for precise filtration, effectively removing impurities, particles, bacteria, and even viruses from water sources. Additionally, nanomembranes can selectively target specific pollutants, making them ideal for removing heavy metals, organic contaminants, and emerging pollutants that pose significant environmental and health risks. In desalination, nanomembranes play a crucial role in efficiently removing salts and minerals from seawater or brackish water, addressing freshwater scarcity challenges in coastal regions. This breakthrough technology has the potential to revolutionize desalination processes and provide a sustainable source of freshwater. The integration of nanomembranes with advanced technologies, such as nanocatalysts and sensors, enables real-time monitoring and targeted pollutant degradation, further enhancing the efficiency and effectiveness of water remediation processes. The environmental benefits of nanomembranes are evident, as their implementation can reduce the energy consumption and environmental footprint associated with conventional water treatment methods. Their high selectivity and efficiency translate into decreased chemical and energy usage, making them an environmentally friendly choice for water purification. Despite the significant progress made in nanomembrane research, challenges remain. The scalability and cost-effectiveness of large-scale production require further attention. Continuous efforts are needed to optimize manufacturing processes, reduce production costs, and make nanomembrane technology economically viable for widespread implementation. Additionally, as nanomaterials are involved in their fabrication, it is crucial to assess and address any potential environmental and health impacts associated with their use. The responsible disposal of used nanomembranes and the development of sustainable, recyclable materials are essential aspects to be considered in the future. Nanomembranes represent a promising frontier in water remediation. Their exceptional capabilities in selective filtration, desalination, and contaminant removal make them valuable assets in ensuring access to clean and safe water. Continued research and development, along with collaborations between academia, industry, and regulatory bodies, will be pivotal in harnessing the full potential of nanomembranes for addressing global water challenges. Embracing this cutting-edge technology can contribute significantly to achieving water sustainability and safeguarding water resources for generations to come.

## Data Availability

Not applicable.
